# Supplementary material for: Schizophrenic Psychosis Symptoms in a Background of Mild-To-Moderate Carnitine Palmitoyltransferase II Deficiency: A Case Report
Source: Reports (MDPI). Author manuscript; Available in PMC 2026 Mar 16. (PMC12989197; doi:10.3390/reports3040031)
Supplement: Supplementary material [file NIHMS2149886-supplement-Supplementary_material.zip › reports-934184 supplementary final/WICKRAMASEKARA-SUPPLEMENT_REV_Clean-FINAL.pdf]

SUPPLEMENTARY MATERIALS AND METHODS

Human participants

Participants in this study were recruited by Dr. Venkata Kolli under Creighton University IRB-approved protocol #1172777 in compliance with all relevant federal, state and local regulations and the Declaration of Helsinki. Consented participant DNA was collected from saliva using the Oragene OGR-500 collection kit (DNA Genotek; Ottawa, Ontario, Canada) and extracted using the prepIT•L2P (DNA Genotek) protocol as per manufacturer's instructions. DNA was quantified by NanoDrop (Thermo Fisher; Waltham, MA) and Qubit 2.0 Broad Range (BR) dsDNA kit (Thermo Fisher) prior to sequencing.

Whole-exome sequencing (WES)

WES and variant calling were performed on de-identified samples at BGI (BGI Group; Shenzhen, Guangdong, China). The qualified genomic DNA sample was randomly fragmented by Covaris technology and the size of the library fragments was mainly distributed between 150bp and 250bp. The end repair of DNA fragments was performed, and an "A" base was added at the 3'-end of each strand. Adapters were then ligated to both ends of the end repaired/dA tailed DNA fragments for amplification and sequencing. Size-selected DNA fragments were amplified by ligation-mediated PCR (LM-PCR), purified, and hybridized to the exome array for enrichment. Non-hybridized fragments were then washed out. Captured products were then circularized. The rolling circle amplification (RCA) was performed to produce DNA Nanoballs (DNBs). Each resulting qualified captured library was then loaded on BGISEQ sequencing platforms, and we performed high-throughput sequencing for each captured library to ensure that each sample met the desired average sequencing coverage. Sequencing-derived raw image files were processed by BGISEQ basecalling Software for base-calling with default

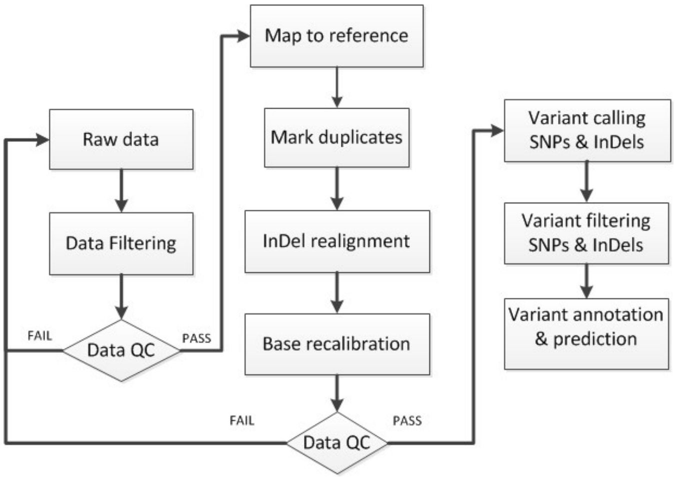

Bioinformatics analysis overview. The bioinformatics analysis began with sequencing data (raw data from the BGISEQ machine). First, the clean data was produced by data filtering on raw data. All clean data from each sample were mapped to the human reference genome using (GRCh37/hg19) using Burrows-Wheeler Aligner (BWA) [3] software. To ensure accurate variant calling, we followed recommended Best Practices for variant analysis with the Genome Analysis Toolkit (GATK, https://www.broadinstitute.org/gatk/guide/best-practices). Local realignment around InDels and base quality score recalibration were performed using GATK [1,2] with duplicate reads removed by Picard tools (http://broadinstitute.github.io/picard/). The sequencing depth and coverage for each individual were calculated based on the alignments. "Low confidence" SNPs were removed before variant calling using GATK HaplotypeCaller (v3.6). After that, a hard-filtering method was applied to get high-confidence variant calls. The SnpEff tool (http://snpeff.sourceforge.net/SnpEff_manual.html) was applied to annotate the variants.

parameters and the sequence data of each individual was generated as paired-end reads, which was defined as "raw data" and stored in FASTQ format for downstream data analysis.

<u>Data analysis</u>

Firstly, in order to decrease noise of the raw sequencing data, data filtering was done, which included: (1) Removing reads containing sequencing adapter; (2) Removing reads whose low-quality base ratio (base quality less than or equal to 5) is more than 50%; (3) Removing reads whose unknown base ('N' base) ratio is more than 10%. Statistical analysis of data and downstream bioinformatics analysis were performed on this filtered, high-quality data, referred to as the "clean data" used for variant calling.

All “clean” reads were aligned to the human reference genome (GRCh37/hg19) using Burrows-Wheeler Aligner (BWA V0.7.15) using the BWA-MEM method. We performed mapping for each lane separately and added the read group identifier into the alignment files. Code for these steps has been provided below (and throughout this document) in blue text.

```
bwa mem -M -R 'read_group_tag' hg19.fasta read1.fq.gz read2.fq.gz > aligned_reads.sam
```

Here the 'read_group_tag' was provided, e.g., '@RG\tID:GroupID\tSM:SampleID\tPL:illumina\tLB:libraryID'.

Picard-tools (v2.5.0) was used to sort the SAM files by coordinate and to convert them to BAM files.

```
java -jar picard-tools-2.5.0/picard.jar SortSam I=aligned_reads.sam
O=aligned_reads.sorted.bam SORT_ORDER=coordinate
```

The same DNA molecules can be sequenced several times during the sequencing process. The resulting duplicate reads are not informative and should not be counted as additional evidence for or against a putative variant. The Genome Analysis Toolkit (GATK), therefore, can ignore them in later analyses. Picard tools (v2.5.0) was used to mark these duplicates.

```
java -jar picard-tools-2.5.0/picard.jar MarkDuplicates \
  I=aligned_reads.sorted.bam \
  O=aligned_reads.sorted.dedup.bam METRICS_FILE=metrics.txt
 java -jar BuildBamIndex.jar I=aligned_reads.sorted.dedup.bam
```

Insertion/deletion (InDel) alignment is notoriously difficult by default pipeline parameters. A realignment step identifies the most consistent placement of the reads relative to the InDel in order to clean up artifacts. This occurs in two steps: first the program identifies intervals that need to be realigned, then, in the second step, it determines the optimal consensus sequence and performs the actual realignment of reads. The use of known “gold standard” InDels from the 1000 Genomes project assist with realignment.

```
java -jar GenomeAnalysisTK.jar -T RealignerTargetCreator \
  -R hg19.fasta \
  -o indels_religner.intervals \
```

```
  -known 1000G_phase1.indels.hg19.vcf \
  -known Mills_and_1000G_gold_standard.indels.hg19.vcf

java -jar GenomeAnalysisTK.jar -T IndelRealigner \
  -R hg19.fasta \
  -I aligned_reads.sorted.dedup.bam \
  -targetIntervals indels_religner.intervals \
  -known 1000G_phase1.indels.hg19.vcf  \
  -known Mills_and_1000G_gold_standard.indels.hg19.vcf \
  -o aligned_reads.sorted.dedup.realigned.bam
```

The variant calling method used heavily relied on the base quality scores in each sequence read. Various sources of systematic error from sequencing machines leaded to over- or under-estimated base quality scores. The BQSR step (below) was necessary to get more accurate base qualities, which in turn improved the accuracy of variant calls. The following commands were used to do this step.

```
java -jar GenomeAnalysisTK.jar -T BaseRecalibrator \
  -R hg19.fasta \
  -I aligned_reads.sorted.dedup.realigned.bam \
  -knownSites dbsnp_138.hg19.vcf \
  -knownSites Mills_and_1000G_gold_standard.indels.hg19.vcf \
  -knownSites 1000G_phase1.indels.hg19.vcf \
  -o recal.table

java -jar GenomeAnalysisTK.jar -T PrintReads \
  -R hg19.fasta \
  -I aligned_reads.sorted.dedup.realigned.bam \
  -BQSR recal.table -o aligned_reads.sorted.dedup.realigned.recal.bam
```

By definition, whole exome sequencing data does not cover the entire reference genome, so variant calling can be restricted to just both the target regions and their flanking regions (extending 200bp towards both sides of each target region). This list of regions was provided in a BED file. The HaplotypeCaller of GATK (v3.6) was used to call both SNPs and InDels simultaneously via local de-novo assembly of haplotypes in regions showing signs of variation. HaplotypeCaller was specifically designed to identify germline variants in diploid samples and is considered a “gold standard” for this application [4]. In brief, for each sample, potential variant

bases are identified using a De Bruijn-like graph approach and genotype likelihoods are calculated using a PairHMM algorithm. A Bayes' rule is applied to each variant likelihood, given the read data, to calculate the genotype (heterozygous or homozygous) [1]. The raw variation set containing all potential variants was outputted as a VCF file.

```
java -jar GenomeAnalysisTK.jar -T HaplotypeCaller \
  -R hg19.fasta --genotyping_mode DISCOVERY \
  -I aligned_reads.sorted.dedup.realigned.recal.bam \
  -L CallVariantRegion/ex_region.sort.bed \
  -o raw_variants.vcf -stand_call_conf 30 -stand_emit_conf 10 -minPruning 3
```

It is extremely important to apply filtering methods to a raw variation set containing both SNPs and InDels in order to move on to downstream analyses with the highest-quality call set possible. The following hard-filtering methods were used on this dataset. First, the SNPs and InDels were separated into two call sets. Secondly, independent filtering parameters were applied to filter SNPs and InDels, respectively. The SNPs and InDels marked as "PASS" in the output VCF file were considered the high confidence variation set. The commands for each step are the following.

```
java -jar GenomeAnalysisTK.jar -T SelectVariants \
  -R hg19.fasta \
  -V raw_variants.vcf -selectType SNP \
  -o raw_snps.vcf
java -jar GenomeAnalysisTK.jar -T SelectVariants \
  -R hg19.fasta \
  -V raw_variants.vcf -selectType INDEL \
  -o raw_indels.vcf
```

Hard filtering for SNPs. The adjustable filtering parameters for SNPs were QualByDepth(QD, the variant confidence divided by the unfiltered depth of non-reference samples), FisherStrand(FS, Phred-scaled p-value using Fishers Exact Test to detect sequencing strand bias in the reads), RMSMappingQuality(MQ, Root Mean Square of the mapping quality of the reads across all samples), MappingQualityRankSumTest (MQRankSum, u-based z-approximation from the Mann-Whitney Rank Sum Test for mapping qualities, only for heterozygous calls), ReadPosRankSum(u-based z-approximation score from the Mann-Whitney Rank Sum Test for the distance from the end of the read for reads with the alternate allele, only for heterozygous calls).

```
java -jar GenomeAnalysisTK.jar -T VariantFiltration \
  -R hg19.fasta -V raw_snps.vcf \
```

```
  --filterExpression "QD<2.0 || FS>60 || MQ<40 || MQRankSum<-12.5 || ReadPosRankSum<-8.0" \
  --filterName "LowConfident" \
  -o filtered_snps.vcf
```

Hard filtering for InDels. The adjustable filtering parameters for InDels were QualByDepth(QD, the variant confidence divided by the unfiltered depth of non-reference samples), FisherStrand(FS, Phred-scaled p-value using Fishers Exact Test to detect sequencing strand bias in the reads), ReadPosRankSum(u-based z-approximation score from the Mann-Whitney Rank Sum Test for the distance from the end of the read for reads with the alternate allele, only for heterozygous calls)

```
java -jar GenomeAnalysisTK.jar -T VariantFiltration \
  -R hg19.fasta -V raw_indels.vcf \
  --filterExpression "QD < 2.0 || FS > 200 || ReadPosRankSum < -20" \
  --filterName "LowConfident" \
  -o filtered_indels.vcf
```

After high-confident SNPs and InDels were identified, the SnpEff tool (http://snpeff.sourceforge.net/SnpEff_manual.html) was applied to perform:

(a) gene-based annotation: identify whether SNPs or InDels cause protein coding changes and the amino acids that are affected.

(b) filter-based annotation: identify variants that are reported in dbSNP v141, or identify the subset of variants with minor allele frequency (MAF) <1% in the 1000 Genomes Project, or identify subset of coding non-synonymous SNPs with SIFT score<0.05, or find intergenic variants with GERP++ score>2, or many other annotations on specific mutations.

Web Resources:

The URLs for data presented herein and data format details are as follows:

UCSC build hg19, {http://hgdownload.cse.ucsc.edu/goldenPath/hg19/bigZips}

RefGene database for hg19, {http://hgdownload.cse.ucsc.edu/goldenPath/hg19/database/refGene.txt.gz}

dbSNP, {http://www.ncbi.nlm.nih.gov/snp}

GATK database for GRCh37(b37), {ftp://gsapubftp-anonymous@ftp.broadinstitute.org/bundle/b37}

1000 Genomes Project database, ftp://ftp-trace.ncbi.nih.gov/1000genomes/ftp/release}

SAM/BAM file format, Sequence Alignment/Map Format Specification {http://samtools.github.io/hts-specs/SAMv1.pdf}

VCF format, {http://www.1000genomes.org/wiki/analysis/vcf4.0}

Variant validation

Sanger sequencing (Genewiz; South Plainfield, NJ) was used to confirm variants of interest from WES following manual filtering using the Integrated Genomics Viewer [5,6]. Primers were designed using BatchPrimer3 v1.0 and synthesized by IDT (Coralville, IA). DNA was amplified using 2X PCR Master Mix (Roche) with a primer concentration of 0.5 µM. PCR products were confirmed by 2% agarose gel electrophoresis and prepared for sequencing using ExoSAP-IT PCR Product Cleanup Reagent (Thermo Fisher). Samples were quantified using the Qubit 2.0 High Sensitivity (HS) dsDNA kit (Thermo Fisher).

SUPPLEMENTARY TABLES

Supplementary Table 1. Sequencing metrics for family 10000.

Included in the attached spreadsheet.

Supplementary Table 2. Variant summary for family 10000.

Included in the attached spreadsheet.

Supplementary Table 3. Coding SNPs identified in the proband.

Included in the attached spreadsheet.

Supplementary Table 4. Coding INDELs identified in the proband.

Included in the attached spreadsheet.

Supplementary Table 5. Inheritance of coding variants in the proband.

Included in the attached spreadsheet.

Supplementary Table 6. Sanger primers for orthogonal validation.

Primer Name	Gene	rs ID	Sequence (5' -> 3')
P001_F	MUTYH	rs34612342	CCCCCTAGCTCCTCTACCAC
P001_R	MUTYH	rs34612342	CCAGTGTGGGTCTCAGAGGT
P002_F	CPT2	rs1799821	TCGGCAGTGTTCTGTCTCTG
P002_R	CPT2	rs1799821	CTCGTAGGTGGCCACTGTCT
P003_F	CPT2	rs1799822	CAACTGGATAGGCTGCAATG
P003_R	CPT2	rs1799822	TAGCACCCACTGGCTACACA
P004_F	APOE	rs440446	TATTACTGGGCGAGGTGTCC
P004_R	APOE	rs440446	ATGGCTTACATCCCAGTCCA
P005_F	APOE	rs429358	GATGGACGAGACCATGAAGG
P005_R	APOE	rs429358	CACCTGCTCCTTCACCTCGT
P006_F	DBH	rs74853476	GCAGCCTTCATGTACAGCAC
P006_R	DBH	rs74853476	AGGACCATGGAAAGCATGTC
P007_F	DBH	rs1611115	CGTTCGTGCAAAGACACAGT
P007_R	DBH	rs1611115	CTGCTCCCCTGTCTCTGAAG

Supplementary Table 7. Population frequencies of rs1799821 from the ExAC database.

Population	Allele Count	Allele Number	Number of Homozygotes	Allele Frequency
East Asian	6325	8634	2331	0.7326
European (Non-Finnish)	36217	66604	9849	0.5438
European (Finnish)	3504	6596	933	0.5312
Other	408	908	107	0.4493
Latino	4947	11528	1062	0.4291
African	2941	10370	451	0.2836
South Asian	4305	16500	613	0.2609
Total	58647	121140	15346	0.4841

Supplementary Table 8. Population frequencies of rs1799822 from the ExAC database.

Population	Allele Count	Allele Number	Number of Homozygotes	Allele Frequency
European (Non-Finnish)	14344	66586	1554	0.2154
Other	136	906	8	0.1501
European (Finnish)	875	6604	64	0.1325
Latino	1395	11524	90	0.1211
South Asian	1657	16452	110	0.1007
East Asian	738	8642	27	0.0854
African	469	10380	9	0.04518
Total	19614	121094	1862	0.162

Supplementary Table 9. Population frequencies of rs74853476 from the ExAC database.

Population	Allele Count	Allele Number	Number of Homozygotes	Allele Frequency
African	10	9524	0	0.00105
European (Non-Finnish)	54	62410	0	0.0008652
Latino	3	10842	0	0.0002767
European (Finnish)	1	5602	0	0.0001785
East Asian	0	8112	0	0
Other	0	830	0	0
South Asian	0	15534	0	0
Total	68	112854	0	0.0006025

Supplementary Table 10. Coding SNPs identified in the sibling.

Included in the attached spreadsheet.

REFERENCES

1. McKenna, A.; Hanna, M.; Banks, E.; Sivachenko, A.; Cibulskis, K.; Kernytsky, A.; Garimella, K.; Altshuler, D.; Gabriel, S.; Daly, M., et al. The Genome Analysis Toolkit: a MapReduce framework for analyzing next-generation DNA sequencing data. *Genome Res* **2010**, *20*, 1297-1303, doi:10.1101/gr.107524.110.
2. DePristo, M.A.; Banks, E.; Poplin, R.; Garimella, K.V.; Maguire, J.R.; Hartl, C.; Philippakis, A.A.; del Angel, G.; Rivas, M.A.; Hanna, M., et al. A framework for variation discovery and genotyping using next-generation DNA sequencing data. *Nat Genet* **2011**, *43*, 491-498, doi:10.1038/ng.806.
3. Li, H.; Durbin, R. Fast and accurate long-read alignment with Burrows-Wheeler transform. *Bioinformatics* **2010**, *26*, 589-595, doi:10.1093/bioinformatics/btp698.
4. Poplin, R.; Ruano-Rubio, V.; DePristo, M.; Fennell, T.J.; Carneiro, M.; Van der Auwera, G.; Kling, D.; Gauthier, L.; Levy-Moonshine, A.; Roazen, D., et al. Scaling accurate genetic variant discovery to tens of thousands of samples. bioRxiv, 2018; 10.1101/201178.
5. Robinson, J.T.; Thorvaldsdottir, H.; Winckler, W.; Guttman, M.; Lander, E.S.; Getz, G.; Mesirov, J.P. Integrative genomics viewer. *Nat Biotechnol* **2011**, *29*, 24-26, doi:10.1038/nbt.1754.
6. Thorvaldsdottir, H.; Robinson, J.T.; Mesirov, J.P. Integrative Genomics Viewer (IGV): high-performance genomics data visualization and exploration. *Brief Bioinform* **2013**, *14*, 178-192, doi:10.1093/bib/bbs017.
